# Mechanisms of Epithelial-Mesenchymal Transition and Prevention of Dispase-Induced PVR by Delivery of an Antioxidant αB Crystallin Peptide †

**DOI:** 10.3390/antiox11102080

**Published:** 2022-10-21

**Authors:** Iori Wada, Parameswaran G Sreekumar, Christine Spee, Andrew J MacKay, Michael Ip, Ram Kannan

**Affiliations:** 1 Doheny Eye Institute, Pasadena, CA 91103, USA; 2 Department of Pharmacology and Pharmaceutical Sciences, School of Pharmacy, University of Southern California, Los Angeles, CA 90089, USA; 3 Department of Biomedical Engineering, Viterbi School of Engineering, University of Southern California, Los Angeles, CA 90089, USA; 4 Department of Ophthalmology, Keck School of Medicine, University of Southern California, Los Angeles, CA 90089, USA; 5 Stein Eye Institute, Geffen School of Medicine, University of California, Los Angeles, CA 90095, USA

**Keywords:** PVR, retinal pigment epithelium, EMT, MET, ROS, mitochondrial function, αB crystallin chaperone peptide, elastin-like polypeptide, nano delivery

## Abstract

Proliferative Vitreoretinopathy (PVR) is a refractory retinal disease whose primary pathogenesis involves the epithelial-mesenchymal transition (EMT) of retinal pigment epithelial (RPE) cells. At present, there is no effective treatment other than surgery for PVR. The purpose of this study was to investigate the effect of αB crystallin peptide (αBC-P) on EMT in PVR. We have previously shown that this peptide is antiapoptotic and regulates RPE redox status. Subconfluent primary human RPE (hRPE) cells were stimulated by TGFβ2 (10 ng/mL) with or without αBC-P (50 or 75 μg/mL) for 48 h and expression of EMT/mesenchymal to epithelial transition (MET) markers was determined. Mitochondrial ROS (mtROS) generation in hRPE cells treated with TGFβ2 was analyzed. The effect of TGFβ2 and αBC-P on oxidative phosphorylation (OXPHOS) and glycolysis in hRPE was studied. RPE cell migration was also assessed. A PVR-like phenotype was induced by intravitreal dispase injection in C57BL/6J mice. PVR progression and potential therapeutic efficiency of αBC-Elastin-like polypeptides (ELP) was studied using fundus photography, OCT imaging, ERG, and histologic analysis of the retina. αSMA, E-cadherin, Vimentin, Fibronectin and, RPE65, and CTGF were analyzed on Day 28. Additionally, the amount of VEGF-A in retinal cell lysates was measured. The EMT-associated αSMA, Vimentin, SNAIL and SLUG showed a significant upregulation with TGFβ2, and their expression was significantly suppressed by cotreatment with αBC-P. The MET-associated markers, E-cadherin and Sirt1, were significantly downregulated by TGFβ2 and were restored by αBC-P. Incubation of hRPE with TGFβ2 for 24 h showed a marked increase in mitochondrial ROS which was noticeably inhibited by αBC-ELP. We also showed that after TGFβ2 treatment, SMAD4 translocated to mitochondria which was blocked by αBC-ELP. Mitochondrial oxygen consumption rate increased with TGFβ2 treatment for 48 h, and αBC-P co-treatment caused a further increase in OCR. Glycolytic functions of RPE were significantly suppressed with αBC-P (75 μg/mL). In addition, αBC-P significantly inhibited the migration from TGFβ2 treatment in hRPE cells. The formation of proliferative membranes was suppressed in the αBC-ELP-treated group, as evidenced by fundus, OCT, and H&E staining in dispase-induced PVR in mice. Furthermore, ERG showed an improvement in c-wave amplitude. In addition, immunostaining showed significant suppression of αSMA and RPE65 expression. It was also observed that αBC-ELP significantly reduced the expression level of vimentin, fibronectin, and CTGF. Our findings suggest that the antioxidant αBC-P may have therapeutic potential in preventing PVR by reversing the phenotype of EMT/MET and improving the mitochondrial function in RPE cells.

## 1. Introduction

Proliferative vitreoretinopathy (PVR) is characterized by fibrosis and scarring of the retina, which is the primary cause of retinal reattachment failure in 5–10% of cases of retinal detachment [1]. There is no effective treatment other than surgery at this time, and repeated vitreoretinal surgery carries the risk of increased inflammation and fibrosis of the retina. Therefore, it is important to develop new molecular targeting therapies based on the exact pathogenesis of PVR. Clinically, the leading cause is the proliferation of primary retinal pigment epithelial (RPE) cells with myofibroblastization, epithelial-to-mesenchymal transition (EMT) within the retinal hyaline and on the inner and outer retinal surfaces, and the formation and shrinkage of cellular fibrous membranes derived from RPE cells [2,3,4]. We and others have investigated mechanism of EMT development in the eye and RPE [5,6,7]. However, the therapeutic intervention based on mechanisms of EMT to mesenchymal to epithelial transition (MET) has not been addressed in detail. Our present study investigates the role of the 20-mer antioxidant αB-crystallin (αBC) peptide in vitro and in vivo experimental models of PVR. 

αB-crystallin (αBC) is a critical member of the small heat shock protein family and is distributed in the lens, cornea, iris, ciliary body, RPE cells, optic nerve, and extraocular tissues [8,9,10]. Our laboratory has a long-standing interest in the metabolic role of αBC in RPE and in preventing oxidatively stressed RPE/retina in age-related macular degeneration (AMD) and related diseases [11,12,13,14]. We showed that αBC is secreted by RPE cells primarily from the apical domain [15]. RPE cells lacking αBC were found to be vulnerable to oxidative and endoplasmic reticulum stress, and RPE cells overexpressing αBC were resistant to apoptotic cell death [16,17,18]. Other significant findings include novel roles of αBC in ocular angiogenesis and subretinal fibrosis [5,19]. 

Our laboratory has been studying the role of peptides derived from the chaperone moiety of parent αB crystallin protein and we have identified a 20-mer peptide (αBC-P) to have multi-faceted properties, the important among them being a key antioxidant that increases glutathione in RPE cells [12,14]. Our studies also revealed that the recombinantly fused αB crystallin peptide with elastin-like polypeptides (ELPs) exhibited antiapoptotic properties and protected RPE cells and retina in experimental models of AMD [13,20].

Transforming growth factor beta 2 (TGFβ2) has been demonstrated to be a powerful inducer of EMT in RPE cells, and the involvement of EMT in PVR processes has also been extensively explored [21,22]. It has been determined that enhanced collagen synthesis and deposition in PVR eyes are caused in part by elevated TGFβ2 levels that have been identified in the vitreous of PVR patients [22,23,24]. TGFβ signaling induces EMT by activating either Smad or non-Smad pathways [25,26]. The Smad-dependent pathway recruits R-smad (Smad2 and Smad3) when TGFβ- receptor Type I and Type II are activated [26]. The transcription of several important genes linked to EMT is induced when activated R-Smad proteins translocate into the nucleus and form a complex with the common Smad (co-Smad) protein Smad4 [25,27]. TGFβ regulates EMT in the Smad-independent pathway by interacting with the JNK/p38-MAPK pathway in an IRE1-dependent manner [26,28]. We and others have demonstrated that TGFβ-induced EMT increases smad4 levels in the nucleus [5,25], but it is unknown if smad4 is translocated to RPE mitochondria or whether the αBC peptide has an impact on mitochondrial function and bioenergetics.

Dispase, a neutral protease derived from *Bacillus polymyxa*, can be utilized as an intravitreal injectable substance to induce PVR. Dispase has been used more frequently recently to induce the PVR model in the eyes of mice and rabbits [27,28,29,30]. According to earlier research, intravitreal dispase injection in mice resulted in the presence of neutrophils in the anterior chamber and PVR-like indications in the retinas, but no particular immune reactions were observed [31,32]. Since mice are easy to manipulate genetically, this model has the benefit that it should be very helpful for uncovering PVR risk factors and testing therapeutic molecules.

This study evaluated whether the chaperone peptide of αB crystallin (αBC peptide) can suppress EMT in RPE cells. We also investigated the mitochondrial function of RPE in TGF-β-induced EMT and the role of αBC peptide in this process. The mechanisms of PVR development were studied in a dispase-induced mouse model [28,33]. The role of αBC-ELP peptide in mitigating PVR and the associated mechanisms involving EMT proteins and cytokines was also investigated.

## 2. Materials and Methods

### 2.1. Cell Culture and Treatment

All experiments were conducted in compliance with the Declaration of Helsinki and ARVO guidelines. Human retinal pigment epithelium (hRPE) cells were isolated from human fetal eyes (gestational age 16–18 weeks) obtained from Novogenix Lab (Los Angeles, CA, USA). Primary cultures of hRPE cells were established as described previously [34]. All experiments used second to fourth passage hRPE cells. In brief, the hRPE cells were grown in Dulbecco’s modified Eagle medium (DMEM, Fisher Scientific, Pittsburgh, PA, USA) with 10% fetal bovine serum (FBS, Laguna Scientific, Laguna Niguel CA, USA). RPE cells that were sub-confluent (about 60% confluency in 3% FBS at the time of treatment) were used in studies with TGFβ2 with or without αBC-peptide (αBC-P) (50 and 75 μg/mL). The peptide sequences were DRFSVNLDVKHSPEELKVK for αBC-P; and DLPLKKNVEDKFHRSFVESV for and scrambled peptide sequence (LifeTein, LLC, Hillsborough, NJ, USA) and the peptides were >98% pure. 

### 2.2. RNA Isolation and Real-Time Quantitative RT-PCR

Total RNA was isolated from cells using an RNA extraction kit (Qiagen, Valencia, CA, USA), and RT-PCR was performed as described previously [5]. Gene expression levels were normalized relative to GAPDH mRNA and reported as fold-change over control. The sequences of primers used are presented in Table 1. Relative multiples of change in mRNA expression were determined by calculating 2^−ΔΔCT^. Results are reported as the mean difference in relative multiples of change in mRNA expression ± SEM.

### 2.3. Western Blot Analysis

Protein was extracted from cells and posterior eyecup without conjunctiva, sclera, and muscle tissue with RIPA buffer containing protease inhibitor. The concentration of soluble protein was measured using BSA as standard (SpectraMax iD5, Molecular Devices, Sunnyvale, CA, USA). Equal amounts of protein (30 μg) were resolved on TGX-precast gels (Bio-Rad Laboratories Inc., Hercules, CA, USA) and transferred to PVDF blotting membranes (Millipore, Billerica, MA, USA). Membranes were probed with respective primary antibodies overnight at 4 °C (The antibodies and dilutions used are listed in Table 2). After incubation with the appropriate secondary antibodies (Vector Laboratories, Burlingame, CA, USA), protein bands were visualized by a chemiluminescence (ECL) detection system (Bio-Rad Laboratories Inc., Hercules, CA, USA). Equal protein loading was confirmed with GAPDH. Protein band intensity was measured by Image J software (Version 1.51 23 April 2018 and URL accessed on 12 April 2018) (https://imagej.nih.gov/ij/). The quantification indicates the relative amounts as a ratio of each protein band to the appropriate loading control and expressed arbitrary units [35].

### 2.4. Detection of Mitochondrial Superoxide with MitoSOX

The production of superoxide in mitochondria was visualized with MitoSOX (Thermo Fisher Scientific, Waltham, MA, USA). Subconfluent hRPE cells grown on four-well chamber slides were treated with TGFβ2 (10 ng/mL) alone or cotreated with αBC- ELP for 24 h. Before the termination of treatment, cells were incubated with 5 μM MitoSOX for 15 min at 37 °C. Cells were washed in PBS, fixed with 3.7% PFA for 15 min, washed, mounted with 4′,6-diamidino-2phenylindole (DAPI: Vector Laboratories, Burlingame, CA, USA), and viewed under a laser confocal microscope (LSM 710: Zeiss, Thornwood, NY, USA).

### 2.5. Measurement of Cellular Respiration

Mitochondrial bioenergetics was studied by measuring oxygen consumption rate (OCR) of RPE cells with a Seahorse XFe96 Analyzer (Agilent, Santa Clara, CA, USA) [35]. Sub-confluent hRPE cells were treated for 48 h with either TGFβ2 (10 ng/mL) or/with αBC-P (50 and 75 μg/mL). In order to start the assays, a 175 μL XF assay media was substituted for the growth medium. XF assay medium includes glucose (25 mM), sodium pyruvate (1 mM), and glutamine (2 mM). The inhibitor concentrations were 1.5 M oligomycin (ATP-Synthase inhibitor), 1.5 M carbonyl cyanide (4-(trifluoromethoxy)-phenylhydrazone) (FCCP, mitochondrial membrane depolarizer), and 0.5 M rotenone (complex 1 inhibitor) and antimycin A (complex 3 inhibitors). We assessed basal respiration, maximal respiration, OCR-linked ATP generation, spare respiratory capacity, non-mitochondrial oxygen consumption, and proton leak. The experiments were repeated three times. The OCR results were expressed as pmol/min/µg protein.

### 2.6. Measurement of Cellular Glycolysis

Glycolysis Stress Test Kit (Agilent, # 103020-100) was used per the standard protocol [36]. Following glucose deprivation, the kit assesses the capacity of the glycolytic pathway. The rate at which the media surrounding the cells becomes more acidic as a result of glycolysis is directly measured by the analyzer and reported as the extracellular acidification rate (ECAR). hRPE cells were seeded in 96-well plates and treated with TGFβ2 (10 ng/mL) for 48 h. The ECAR was measured (17–20 wells/experimental conditions) before and after sequential injections of glucose (10 mM), oligomycin (1 M), and 2-Deoxy-D-glucose (2-DG; 50 mM) in in order to investigate the glycolytic potential of cells after the treatment period. The results were presented as mpH/min/µg protein.

### 2.7. Cell Migration Assay

To analyze cell migration, hRPE cells were seeded on ninety-six well plates at a density of 5 x 10^4^ cells per well. A wound was made by scraping hRPE cells monolayer with WoundMaker^TM^ (Essen BioScience, Inc., Ann Arbor, MI, USA). Cells were treated with either TGFβ2 (10 μg/mL) or with TGFβ2 + αBC-P, and images were analyzed using Incucyte SX5 Live-Cell Analysis System (Essen BioScience, Inc., Ann Arbor, MI, USA) at zero hours, 6, 12, 18, and 24 h after scratch. The percentage of hRPE cell migration was obtained with Image J software (NIH). At least three fields (magnification 4×) of view per treatment of six independent experiments were quantified. 

### 2.8. Dispase-Induced PVR in Mouse

All animal studies were approved by the University of California, Los Angeles, Animal Research Committee and adhere to the Doheny Eye Institute Animal Statement. All experimental procedures on the animals were performed according to the ARVO Statement for the Use of Animals in Ophthalmic and Vision Research. We used the dispase model of PVR in mice according to published procedures [28,33]. Male C57BL/6J mice (6–8 weeks old) that do not carry the rd8 and Pde6brd1 mutations were purchased from the Jackson Laboratory (The Jackson Laboratory, Bar Harbor, ME, USA). Intravitreal injections of dispase (1 µL, 5 mg/mL stock) were performed under a binocular surgical microscope (OMS-90 Operation Microscope, Topcon Healthcare, Oakland, NJ, USA) using a 10 μL Hamilton syringe. Since regular saline has been known to cause retinal degeneration, we used cell culture grade sterile PBS as controls in all of our studies [36]. Dispase-administered animals were given either αBC-Elastin-like polypeptides (Cry SI, αBC-ELP, 1 µL, 218 µM stock) or ELP alone (SI, µL, 218 µM) on days 7 and 14. The ELP preparations used in this study were evaluated for their biochemical properties and chaperone function [13,20]. Twentyeight days later, fundus and optical coherence tomography (OCT) images were taken using the Retinal Microscopic Imaging System (Phoenix Research Labs, Pleasanton, CA, USA) and an ERG using the Celeris system (Diagnosys LLC in Lowell, MA, USA). At the termination of the experiment on Day 28, the eyes were enucleated for histological and immunofluorescence staining analysis. At the termination of the experiment on Day 28, the eyes were enucleated for histological and immunofluorescence staining analysis. 

### 2.9. Immunofluorescence

Retinal cryosections (8 μm) were fixed with methanol for 20 min and washed with PBS. After a 30 min blocking step with 10% goat serum, the tissues were incubated overnight at 4 °C with primary antibodies, anti-rabbit αSMA, anti-rabbit E-cadherin, anti-rabbit RPE65 (1:100 dilution), and incubated with corresponding secondary antibodies (Vector Laboratories Inc., Newark, CA, USA). Images were acquired using Keyence (BZ-X710, KEYENCE, Osaka, Japan). The quantification of the images was carried out as described in a previous report [37]. 

### 2.10. Enzyme-Linked Immunosorbent Assay (ELISA) 

Posterior eyecup without conjunctiva, sclera, and muscle tissue was homogenized in RIPA buffer with protease inhibitor. After centrifugation, supernatants containing the soluble proteins were collected. VEGF protein levels in the supernatant (40 µg protein/well) were determined using an ELISA kit per the manufacturer’s instructions (Quantikine ELISA mouse VEGF; R&D Systems, Minneapolis, MN, USA). Absorbance was measured at 450–570 nm using a SpectraMax iD5 (Molecular Devices, Sunnyvale, CA, USA). Data are represented as pg/mL. 

### 2.11. Statistical Analysis

All data are expressed as mean ± SEM. Statistical analysis was performed using Analysis of Variance (ANOVA), followed by a Tukey-post test (JMP pro software, version 15.1.0; SAS, Inc., Cary, NC, USA). *p* < 0.05 was considered significant.

## 3. Results

### 3.1. αBC-P Suppressed EMT-Associated Genes in hRPE Cells

**Figure 1:**
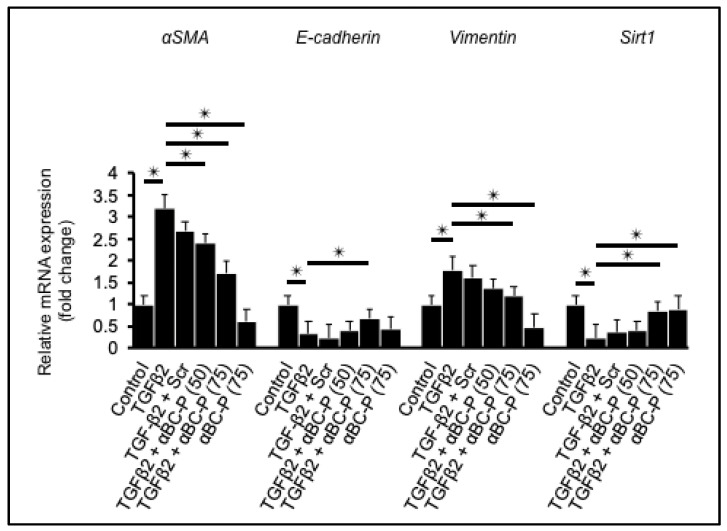
Attenuation of EMT in TGFβ2–induced hRPE by αBC-P. Sub-confluent fetal hRPE cells were treated with TGFβ2 (10 ng/mL) alone or/with Scr. (75 μg/mL) or/with αBC-P (50 and 75 μg/mL) or αBC-P (75 μg/mL) alone for 48 h. αSMA and Vimentin mRNA increased significantly with TGFβ2 treatment, and αBC-P treatment significantly decreased their levels. E-cadherin and Sirt1 mRNA decreased significantly with EMT, and αBC-P treatment significantly increased their levels. Scr: Scrambled peptide, αBC-P: αB crystallin-chaperone peptide. Values are means ± SEM. * *p* < 0.05. *n* = 5.

We examined the expression of EMT- and MET-related markers, αSMA, Vimentin, E-cadherin, and Sirt1. Expression of these EMT-related genes after 48 h in TGFβ2 (10 ng/mL) -treated hRPE cells showed a significant 3-fold and 1.5-fold upregulation of αSMA and Vimentin mRNA levels. On the other hand, MET-related genes after 48 h TGFβ2 treatment showed a significant 3-fold and 4-fold downregulation of E-cadherin and Sirt1 mRNA levels. αBC-P co-treatment significantly (*p* < 0.01 vs. TGFβ2 treated cells) inhibited TGFβ2-induced upregulation of both αSMA and Vimentin at the mRNA levels at 50 and 75 µg/mL concentrations and caused downregulation of both E-cadherin and Sirt1 at the mRNA levels (Figure 1). Data for scrambled αBC-P (75 µg/mL) is also included in the figure. 

### 3.2. αBC-P Suppressed EMT-Associated Proteins in hRPE Cells

**Figure 2:**
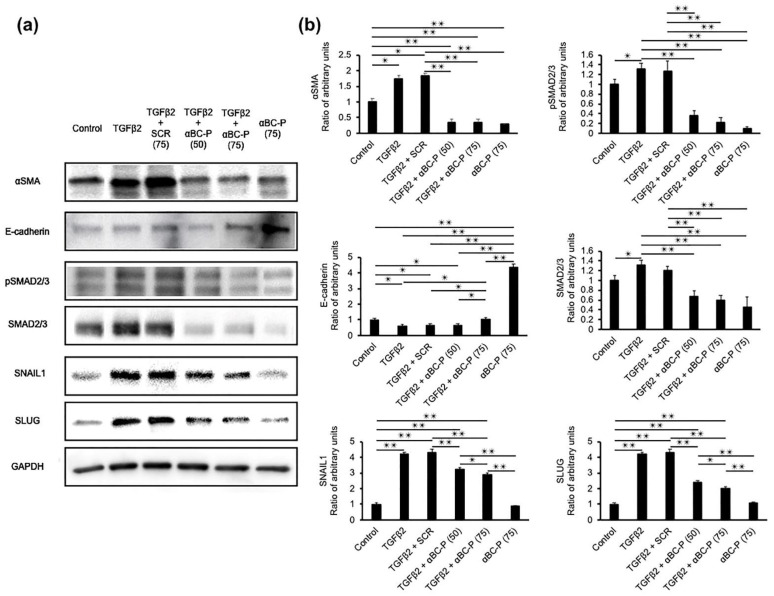

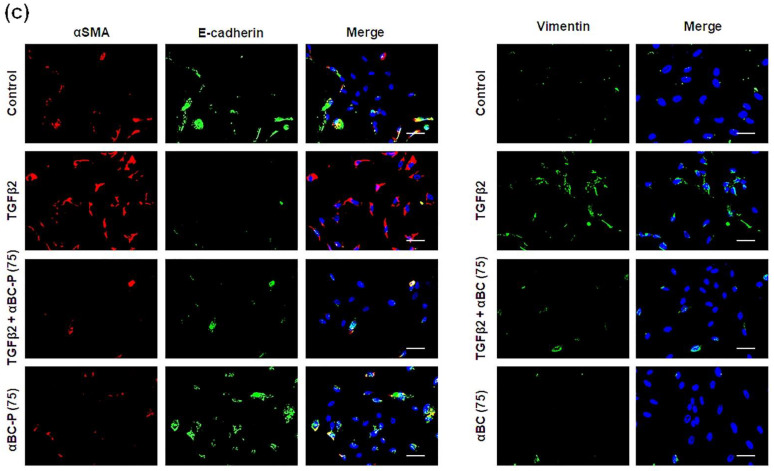
Regulation of EMT- and MET-associated proteins by αBC-P in fetal hRPE cells. (**a**,**b**) Sub-confluent fetal RPE cells were treated with TGFβ2 (10 ng/mL) alone or/with Scr. (75 μg/mL) or/with αBC-P (50 and 75 μg/mL) or αBC-P (75 μg/mL) alone for 48 h. Protein expression was measured by Western blot analysis. EMT-associated proteins (αSMA, pSMAD2/3, SMAD2/3) and transcription proteins SNAIL and SLUG increased significantly with TGFβ2 stimulation, and αBC-P markedly inhibited their expression. Moreover, MET-associated protein (E-cadherin) decreased significantly with TGFβ2 stimulation, and αBC-P markedly increased their expression. (**c**) hRPE cells were cultured in 4 well chamber slides. The cells were treated with TGFβ2 (10 ng/mL) or/and αBC-P (75) for 24 h at 37 °C. After washing, cells were imaged with fluorescence microscope. Scale bar: 50 µm, (**Left** images) Red: αSMA, Green: E-cadherin, Blue: DAPI. (**Right** image) Red: αSMA, Green: E-cadherin, Blue: DAPI. Scr.: Scrambled peptide, αBC-P: αB crystallin-chaperone peptide. Values are means ± SEM. * *p* < 0.05, ** *p* < 0.01. *n* = 3.

We examined the expression of EMT- and MET-related proteins, αSMA, pSMAD2/3, SMAD2/3, and E-cadherin (Figure 2a). Expression of EMT-related proteins after 48 h in TGFβ2 (10 ng/mL)-treated hRPE cells showed a significant upregulation of αSMA, pSMAD2/3, SMAD2/3 protein levels. On the other hand, expression of MET-related protein E-Cadherin after 48 h in TGFβ2 hRPE cells showed significant downregulation. αBC-P co-treatment significantly (*p* < 0.01 vs. TGFβ2 treated cells) inhibited upregulation of αSMA, pSMAD2/3, and SMAD2/3 protein expression, while αBC-P caused a significant upregulation of E-cadherin (Figure 2b). We also investigated the expression of the key transcription factors for EMT, SNAIL and SLUG. SNAIL and SLUG were significantly upregulated in TGFβ2-treated hRPE cells, however, their upregulation was attenuated by co-treatment with αBC-P (*p* < 0.01 vs. TGFβ2-treated cells). Additionally, immunostaining with αSMA, E-cadherin, and Vimentin yielded results that supported the trend shown in Figure 2a,b. The epithelial marker, E-cadherin was restored following co-treatment with TGFβ2 and αBC-P (Figure 2c).

### 3.3. αBC-ELP Treatment Inhibited Mitochondrial ROS Production in hRPE Cells Exposed to TGFβ2

**Figure 3:**
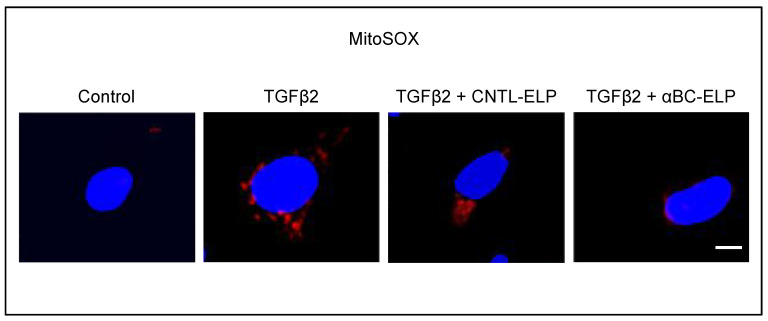
Effect of TGFβ2 on ROS generation and inhibition of ROS by αBC-ELP. hRPE cells were cultured in 4 well chamber slides. Twenty four hour after treatment with TGFβ2 (10 ng/mL) or co-treatment with TGFβ2 (10 ng/mL) and αBC-ELP or co-treatment with TGFβ2 (10 ng/mL) and CNTL-ELP, cells were incubated with 5 µM MitoSox Red (mitochondrial superoxide marker) for 10 min at 37 °C. After washing, cells were imaged with a confocal microscope (ZEISS LSM 710).TGFβ2 at 10 ng/mL (24 h) noticeably increased mitochondrial ROS production in RPE cells and co-treatment with αBC-ELP at 10 µM inhibited TGFβ2 -induced ROS formation. SI is used as negative control (CNTL-ELP). Red: Mitochondrial superoxide, Blue, DAPI, nuclear stain. *Scale bar*: 10 µm.

Reactive oxygen species (ROS) are thought to play a key role in the common fibrotic pathway [38]. We therefore investigated whether TGFβ-2 causes oxidative stress in hRPE cells. TGFβ2 treatment for 24-h-induced mitochondrial superoxide production in hRPE cells. On the other hand, co-treatment with the protein αBC minipeptide fusion protein (αBC-ELP) inhibited the generation of ROS caused by TGFβ-2 (Figure 3). No appreciable cell death was observed in RPE cells treated with varying doses of TGFβ-2 (5–20 ng/mL) for 48 h (data not shown).

### 3.4. αBC-ELP Treatment Inhibited SMAD4 Mitochondrial Translocation in hRPE Cells

**Figure 4:**
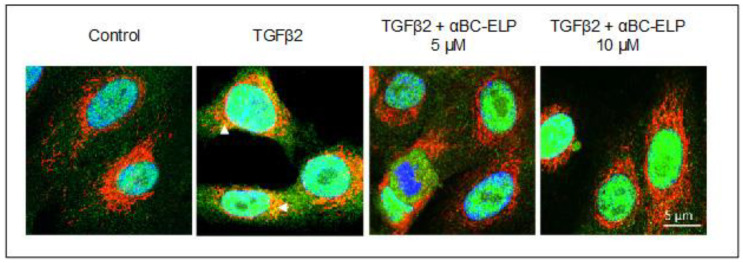
Effect of TGFβ2 on SMAD4 mitochondrial translocation and inhibition by αBC-ELP. hRPE cells were treated with TGFβ2 (10 ng/mL) for 24 h and double stained for mitochondria (Red) and SMAD4 (Green). SMAD4 mitochondrial translocation was observed in TGFβ2 treated cells (white arrow heads) which was inhibited by αBC-ELP at two different doses. *Scale bar*: 5 µm, Green: SMAD4, Red: Mitotracker, Blue, DAPI.

A mitochondrial role of SMAD4 in the pathogenesis of diabetic nephropathy has been reported recently [38]. Therefore, we examined whether TGFβ2 induces SMAD4 mitochondrial translocation in hRPE cells. TGFβ2 treatment for 24 h induced SMAD4 mitochondrial translocation in hRPE cells. On the other hand, co-treatment with αBC-ELP inhibited TGF-β2 -induced SMAD4 mitochondrial translocation (Figure 4).

### 3.5. TGFβ2-Induced Changes in Energy Metabolism and the Role of αBC-P in RPE

**Figure 5:**
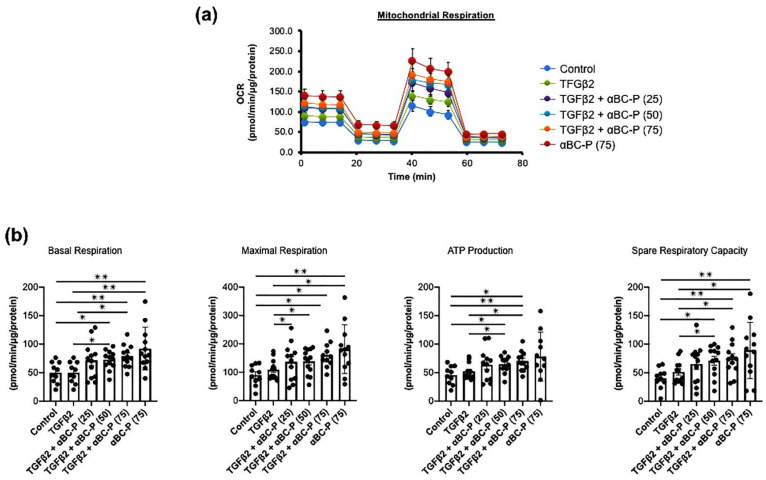
Increased respiration and ATP production in EMT and further activation by αBC-P. Mitochondrial bioenergetics were analyzed using Seahorse XFe96 (**a**). Sub-confluent RPE cells were treated with TGFβ2 (10 ng/mL) alone or/with αBC-P (25, 50, and 75 μg/mL) in DMEM containing 3% FBS for 48 h. TGFβ2-cotreated with αBC-P significantly increased mitochondrial bioenergetic parameters, such as basal respiration, Maximal Respiration, ATP production, and Spare Respiratory Capacity, compared with the TGFβ2-treated group in a dose-dependent manner (**b**). αBC-P: αB crystallin-chaperone peptide. Data normalized by μg/cellular protein. Values are means ± SEM. * *p* < 0.05, ** *p* < 0.01. *n* = 9–15.

We investigated mitochondrial function in TGFβ2 (10 ng/mL)-induced hRPE cells. In comparison to control cells, TGFβ2 treated hRPE cells show higher maximal respiration, ATP generation, and spare respiratory capacity. Co-treatment of hRPE cells with αBC-P significantly increased all mitochondrial bioenergetic parameters in a concentration-dependent manner compared to TGFβ2-treated hRPE cells (Figure 5b). Additionally, identical patterns were seen for non-mitochondrial oxygen consumption and proton leak measurements (Figure S1). These results suggest that αBC-P augments mitochondrial energy metabolism in TGFβ2-treated EMT in RPE cells.

**Figure 6:**
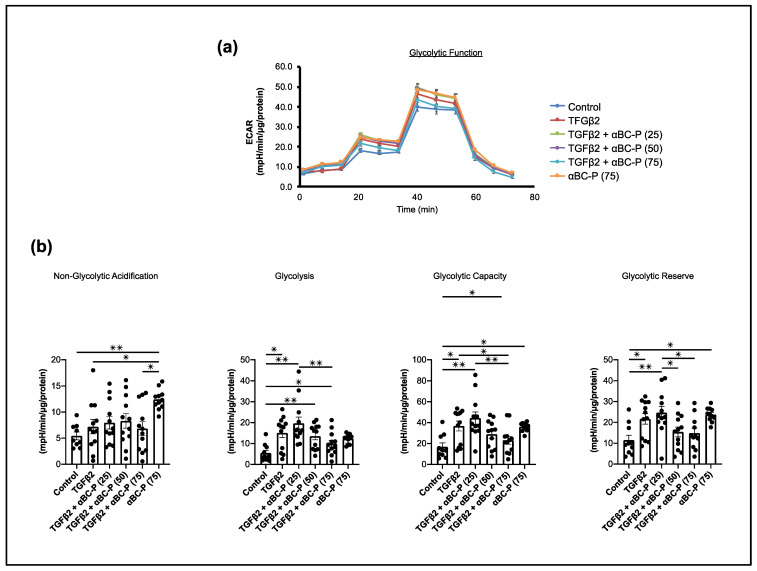
Change in glycolysis in TGFβ-induced EMT in hRPE cells. Real-time monitoring of glycolysis using the Seahorse XFe96 Glycolytic Stress Test Kit for key parameters of the glycolytic function (**a**). TGFβ-induced EMT in hRPE cells significantly increased glycolysis, glycolytic capacity, and glycolytic reserve, but only 75 μg/mL αBC-P significantly suppressed all glycolytic functions (**b**). αBC-P: αB crystallin peptide. N.S.: not significant. Data normalized by total cellular protein. Values are means ± SEM. * *p* < 0.05, ** *p* < 0.01. *n* = 9–15.

We studied changes in glycolysis by measuring the extracellular acidification rate (ECAR) under glucose starvation and subsequent addition. TGFβ2-treated hRPE cells showed increased glycolysis, glycolytic capacity, and glycolytic reserve compared with controls (Figure 6). These results indicate that TGFβ2 treated hRPE cells rely more on glycolysis than control cells. Furthermore, 75 μg/mL αBC-P co-treatment significantly reduced glycolytic function in TGFβ2-induced hRPE cells (Figure 6). Thus, we can conclude that both OXPHOS and glycolysis contribute to TGFβ2-induced mitochondrial metabolism in hRPE. 

### 3.6. αBC-P Inhibits TGFβ2-Induced Migration of hRPE Cells

**Figure 7:**
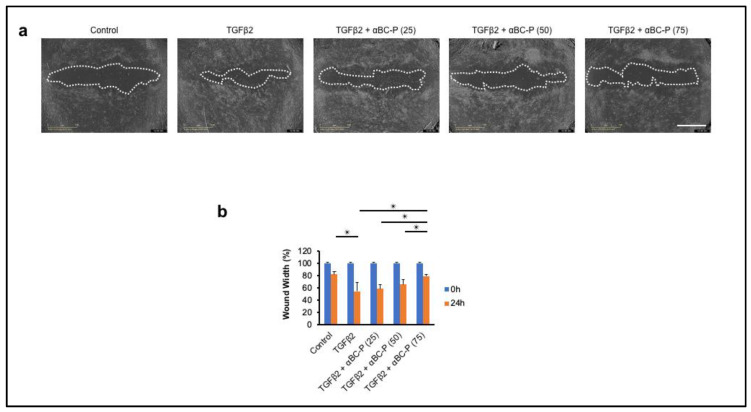
αBC-P inhibits TGFβ2-induced hRPE cell migration. Representative images (**a**) and graphs (**b**) of wound width in RPE cells co-treated with TGFβ2 and αBC-P. hRPE cells were divided into following treatment groups: control, TGFβ2 (10 ng/mL) and/or αBC-P (25, 50 and 75 μg/mL) for 24 h. αBC-P (75 μg/mL) significantly inhibited TGFβ2-treated hRPE cell migration compared to TGFβ2-only treated hRPE cells. αBC-P: αB crystallin-chaperone peptide. *Scale bar*: 1 mm. Values are means ± SEM. * *p* < 0.05. *n* = 8.

The effect of αBC-P on cell migration was evaluated using an in vitro scratch assay [39,40]. hRPE cells were incubated in a 3% FBS medium overnight. Immediately following scratch injury, hRPE cells were treated with TGFβ2 (10 ng/mL) with or without αBC-P (25, 50, and 75 μg/mL). Low (25 μg/mL) and intermediate concentration (50 μg/mL) αBC-P did not inhibit TGFβ2-induced hRPE cell migration, whereas the high dose of αBC-P used (75 μg/mL) significantly inhibited the cell migration (Figure 7). These results suggest that αBC-P may inhibit PVR progression by inhibiting hRPE cell migration.

### 3.7. αBC-P Inhibits Matrix Deposition in Dispase-Induced PVR in Mice

**Figure 8:**
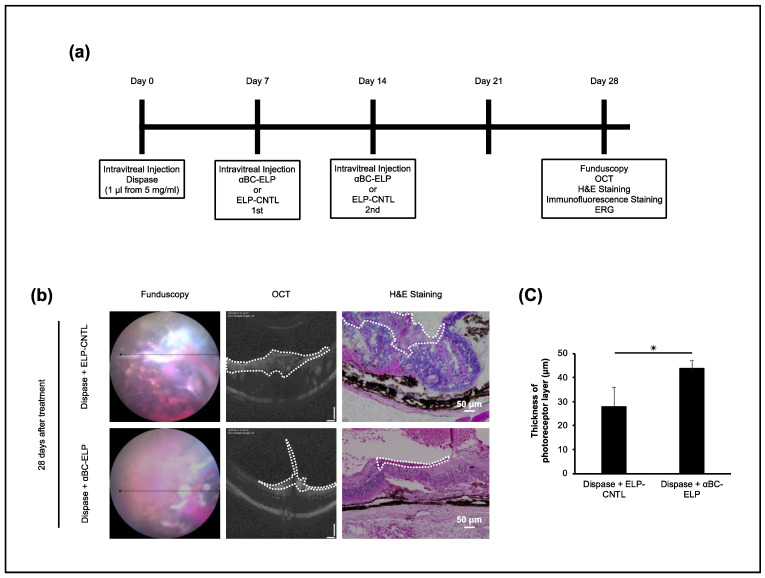
Effect of αBC-ELP in dispase-induced PVR in mouse. Mice were injected with a single intravitreal dose of dispase (1 μL, 5 mg/mL) on day 0. On days 7 and 14, αBC-ELP (CrySI, 1 μL from 218 μM) or Control ELP (SI) was administered intravitreally. On day 28, fundus, OCT images, and ERG data were gathered. At the end of the procedure, mice were euthanized, and retinal sections were processed for H&E and immunostaining. (**a**) Experimental scheme. The ELP preparations for α-BC-ELP and ELP-CNTL are also referred as CrySI and SI, respectively. (**b**) Fundus, OCT images, and H&E staining showed that mice treated with αBC-ELP had less proliferative membrane area and decreased retinal layer structure disruption compared to the dispase + ELP-CNTL group (**b**) The area enclosed by the white dotted line in OCT and H&E staining represents the PVR membrane. (**c**) Quantification of the photoreceptor thickness. αBC-ELP: αB crystallin-Elastin-like peptide. *Scale bar*: 50 μm. * *p* < 0.05, *n* = 5.

We used dispase-induced PVR in mice to investigate the profibrotic effect of αBC-ELP in vivo. In this model, intravitreal injection of dispase induced a PVR-like phenotype characterized by a proliferative membrane or retinal detachment. This resulted in migration of RPE cells to vitreous components, predisposing factors associated with PVR development [28,33,41]. A scheme depicting the experimental protocol is presented in Figure 8a. Our results revealed that Fundus, OCT, and histological analysis showed a less proliferative membrane in dispase with αBC-ELP treated compared with dispase-treated mice (Figure 8b). Moreover, the structure of the retina, RPE and photoreceptor layer (Figure 8c) was better preserved in the dispase + αBC-ELP treatment group. The thickness of the photoreceptor layer was also examined. Compared to the control group, the group that had received αBC-ELP treatment had a considerably intact photoreceptor layer (Figure 8c). However, the functional studies did not support this improvement in photoreceptor structure. These results indicate that αBC-ELP may inhibit the formation of proliferative membranes and prevent retinal damage caused by membrane traction. 

### 3.8. Evidence for αBC-ELP-Induced Improvement in RPE Cell Function by ERG

**Figure 9:**
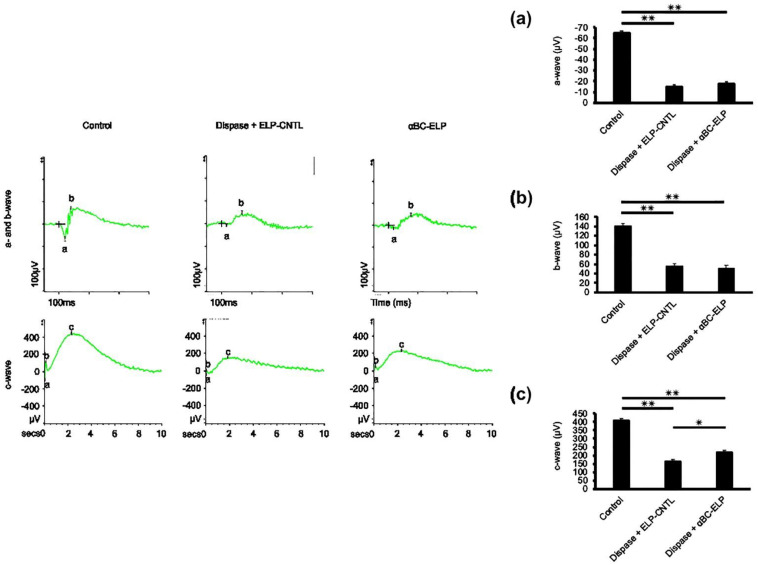
Effect of αBC-ELP on photoreceptor function by ERG. The experimental protocol is shown in Figure 8a. Electroretinogram (ERG) analysis of mice treated with and without αBC-ELP (**a**–**c**). The ‘**a**’—wave represents the hyperpolarization of photoreceptors (**a**), the ‘**b**’ wave represents the second-order neuron response (**b**), and the ‘**c**’ wave represents light-induced activity in the photoreceptors (**c**). Three groups were compared: (1) wild-type with PBS as control, (2) dispase (1 μL, 5 mg/mL) + ELP-CNTL treated, (3) dispase (1 μL, 5 mg/mL) + αBC-ELP (1 μL from 218 μM). While there is no change in (**a**,**b**) waves, (**c**) wave amplitude significantly improved with αBC-ELP. Values are the means ± SE. αBC-ELP: αB crystallin-elastin-like polypeptide. * *p* < 0.05, ** *p* < 0.005. *n* = 3.

We performed ERG analysis in control animals that received PBS and in dispase treated mice with or without αBC-ELP. Dispase treatment caused a reduction in a-wave (photoreceptor rods/cones), b-wave (inner retina, predominantly Muller and ON-bipolar cells), and c-wave (light-evoked responses of the RPE cells) amplitudes. However, ‘a’- and ‘b’-waves had no significant difference between dispase-treated and dispase + αBC-ELP groups (Figure 9a,b). On the other hand, c-wave significantly improved in dispase + αBC-ELP treated when compared to dispase only treated mice. These findings suggest that αBC-ELP may be a promising therapeutic agent in clinical treatment. 

### 3.9. αBC-ELP Inhibits EMT in Dispase-Induced PVR in Mice

**Figure 10:**
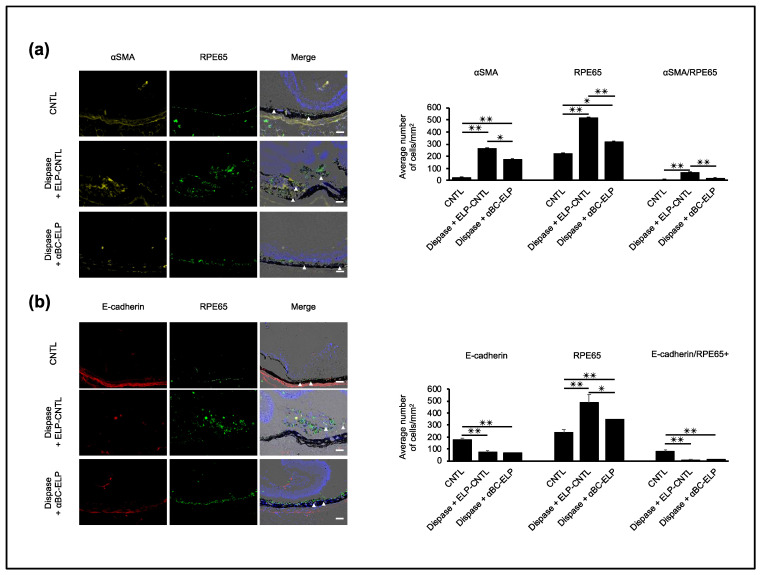
αBC-ELP inhibits EMT in dispase-induced PVR in mice. The experimental protocol is shown in Figure 8a. Double immunofluorescence staining for αSMA or E-cadherin with RPE65 in retinal sections (**a**,**b**). Nuclei are stained blue. White arrowheads indicate co-staining of αSMA/RPE65 and E-cadherin/RPE65, respectively. Quantification of number of positive cells for αSMA, RPE65, E-Cadherin and co-localization is shown in the bar graphs. Values are means ± SEM. N.S.: not significant, αBC-ELP: αB crystallin-Elastin-like peptide. *Scale bar*: 50 μm. * *p* < 0.05, ** *p* < 0.01. *n* = 10.

We used immunostaining to investigate the effect of αBC-ELP on EMT in vivo. αSMA expression was significantly (*p* < 0.05) decreased in mice treated with dispase and αBC-ELP compared to dispase-only treated mice. On the other hand, the expression of E-cadherin showed no significant difference between the two groups (Figure 9b). RPE65 expression that corresponds to RPE migration was significantly (*p* < 0.05) increased in the dispase-treated mice, whereas it significantly decreased with αBC-ELP treatment (Figure 10a). Furthermore, RPE65-expressing cells maintained a monolayered structure in αBC-ELP treated mice as seen in normal eyes (Figure 10a). These results suggest that αBC-ELP may suppress cellular EMT and inhibit the progression of PVR in vivo. 

### 3.10. αBC-ELP Suppressed EMT/MET-Associated Proteins in Dispase-Induced PVR

**Figure 11:**
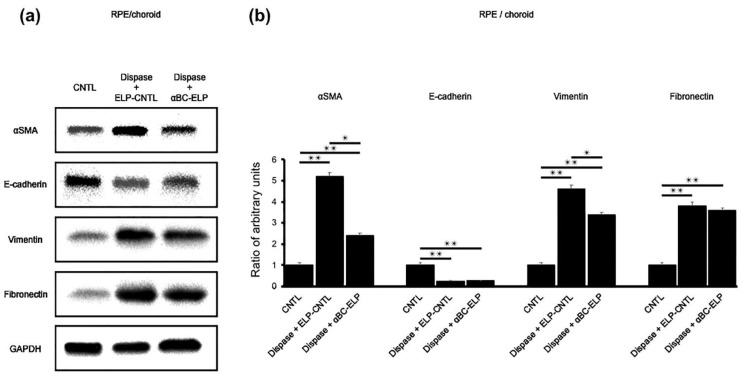
Regulation of EMT- and MET-associated proteins by αBC-ELP in dispase-induced PVR in mice. The experimental protocol is shown in Figure 8a. Protein expression was measured by Western blot analysis (**a**). EMT-associated proteins (αSMA, Fibronectin and Vimentin) significantly increased with dispase treatment, and αBC-ELP treatment significantly inhibited their expression (**b**). MET-associated protein (E-cadherin) had no significant difference with αBC-ELP treatment, and αBC-ELP treatment did not alter the expression (**b**). Values are means ± SEM. N.S.: not significant, αBC-ELP: αB crystallin-Elastin-like peptide. * *p* < 0.05, ** *p* < 0.01. *n* = 3.

We examined EMT-associated protein expression in vivo using protein lysates from the above experiments. The expression of αSMA, Fibronectin and Vimentin EMT-associated proteins, was significantly (*p* < 0.05) decreased with αBC-ELP treatment compared to dispase-only treated mice (Figure 11). On the other hand, the expression of E-cadherin, a MET-associated protein, showed an increasing trend with αBC-ELP treatment compared to dispase alone. However, the increase was not significantly different (Figure 11). These results indicate that αBC-ELP suppresses EMT at the protein level in vivo.

### 3.11. αBC-ELP Suppressed CTGF in Dispase-Induced PVR in Mice

**Figure 12:**
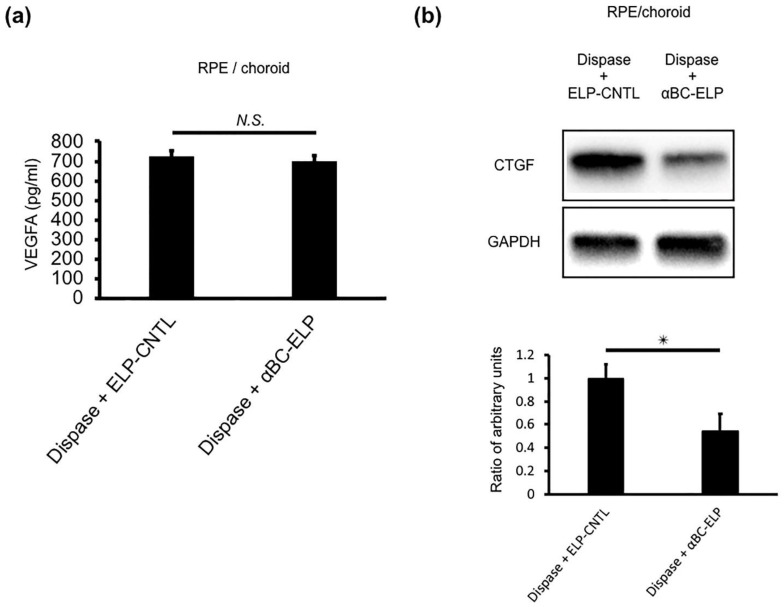
(**a**) VEGFA is measured by ELISA (**a**). CTGF cytokine is analyzed by WB (**b**). CTGF expression was significantly decreased with αBC-ELP treatment. Values are means ± SEM. N.S.: not significant, αBC-ELP: αB crystallin-Elastin-like peptide. * *p* < 0.05. *n* = 3.

We next investigated levels of VEGF and CTGF, two major cytokines associated with PVR [42,43]. First, we examined the VEGF level by ELISA. No significant difference between the dispase-treated group and the dispase + αBC-ELP group (Figure 12a) was found. However, the expression of CTGF was significantly (*p* < 0.05) decreased in the dispase + αBC-ELP treatment group as compared to dispase alone (Figure 12b). These results suggest that αBC-ELP suppresses PVR progression by inhibiting CTGF expression.

## 4. Discussion

Previous reports have shown an essential role for the EMT of RPE cells in PVR development [4,44,45]. In the present study, we provide evidence for the inhibition of RPE-EMT by an antioxidant crystallin peptide in vitro and in vivo. Interestingly, in vitro experimental data showed that αBC-P not only suppresses EMT but may also be involved in the epithelialization of mesenchymal cells, MET. Our data revealed that SMAD4 translocated to mitochondria which could be inhibited by αBC-ELP. However, in in vivo experiments using dispase-induced PVR in mice, a trend toward increased protein level of E-cadherin was observed in dispase + αBC-ELP compared with dispase-induced PVR in mice. 

A brief discussion of the rationale for the experimental design of the in vitro and in vivo studies described in the present work needs to be included. The in vitro experiments in RPE mostly used the free αBC peptide. Our previous work established that αBC peptide protects RPE from cell death by inhibiting caspase 3/7 and augmenting cellular GSH in oxidative stress [12,14]. In particular, we found that a decrease in mitochondrial pool of GSH with oxidative stress and restoration with αBC peptide was critical for RPE survival [46]. The concentrations of αBC peptide used in the present study were in the same range (1.7 to 32 µM) as in our previous studies [12]. We have shown that the peptide is not toxic, taken up by RPE via specific oligopeptide transporters, and is antiapoptotic under these conditions. Despite potential applications across various diseases, major in vivo barriers of small molecular weight peptides, including αBC, need to be considered, particularly in relation to the rapid clearance of peptides. So, in our previous work, we developed a novel nanoparticle delivery system for the αBC-P peptides [13,20]. Using ELP- αBC-P copolymers (CrySI) that have a temperature phase transition, low immunogenicity, and ability to express αBC-P peptides multivalently, we showed RPE protection under oxidative stress as well as retinal protection in models of AMD such as the NaIO_3_-induced retinal degeneration [13]. Further, the mean retention time of αBC-ELP was 3 days compared to αBC peptide alone, which is less than a day [13]. In the present study, we have used one of the known models, namely the dispase-induced murine model of PVR, to investigate the effect of αBC-P [28,33].

This study aimed to discover the role of αBC-P, a short chain (20 amino acid) peptide, in the TGFβ2-induced EMT of RPE. Previous studies in ocular cells reported that TGFβ2 significantly upregulates αB-crystallin [5,6]. We also reported that αB-crystallin causes TGFβ2-induced EMT in RPE cells and that suppression of αB-crystallin by siRNA treatment reduces TGFβ2-induced EMT (mRNA levels and protein expression) [5]. However, the experimental context and in vitro and in vivo experimental models used need to be considered in interpreting the present results, with the αBC-P short chain amino acid peptide showing a different behavior compared to the full-length parent protein. We and others have confirmed in several previous studies that the active chaperone moiety of the αB crystallin protein of the peptide that we have used possesses antiapoptotic, anti-inflammatory, antiangiogenic, and antisenescence properties [6,12,20,35,47].

In the present study, we also found that αBC-P suppressed the SMAD pathway. Previous studies reported that αB-crystallin expression induces phosphorylation of p44/42 MAPK, p38 MAPK, and AKT signaling pathways, and crosstalk between these signaling pathways and Smad-dependent pathways has also been reported [5,6,48,49]. Although the mechanism by which αBC-P directly or indirectly activates Smad-dependent signaling is not clear in the present study, our results suggest a relationship between αB-crystallin expression and the Smad-dependent pathway of EMT in RPE cells. In this study, we found that TGFβ2-treated hRPE cells increased mtROS production and αBC-ELP peptide inhibited mtROS production in vitro. Recently, it has been shown that ROS mediate TGFβ signaling through pathways distinct from the Smad, MAPK, and Rho-GTPase pathways [50,51]. Canonical TGF-β2/SMAD4 signalling involves SMAD4 translocation to the nucleus to regulate gene transcription. However, it has been shown that SMAD4 enters the mitochondria and interacts with the cytochrome c oxidase II protein to trigger apoptosis [50]. However, we could not observe any apoptosis under our experimental conditions. Our results demonstrate that neither untreated cells nor cells cotreated with the αBC-ELP peptide and TGFβ2 had SMAD4 in their mitochondria. In this context, it is interesting to note that coiled-coil-helix-coiled-coil-helix domain-containing protein 2 (CHCHD2), a mitochondrial protein, interacts with Smad4 to repress TGF-β signalling in human-induced pluripotent stem cells [52]. However, more research is required to precisely define the function of mitochondrial SMAD4 in the modulation of mitochondrial complex proteins.

Cellular energy is produced in the mitochondria in the presence of oxygen in the form of adenosine triphosphate (ATP) by oxidative phosphorylation (OXPHOS) and in the absence of anaerobic glycolysis in the cytosol [53]. Our data suggest increased OXPHOS and glycolytic rate in TGFβ2-treated RPE cells. In this study, we report for the first time the relationship between PVR and OXPHOS activity and the glycolytic system: RPE transports glucose to photoreceptor cells, and excessive glucose used by the RPE causes glucose deprivation and cell death in photoreceptor cells [54]. Thus, TGFβ2-induced RPE cells with dysregulated metabolism may not meet the metabolic needs of photoreceptors, inducing RPE cell and photoreceptor cell death, RPE cell proliferation, and abnormal secretion of extracellular matrix [4,54,55].

In our study, we observed the reprogramming of hRPE cells in in vitro experiments. While it is still debatable whether metabolic remodeling is a cause or a consequence of cellular reprogramming, our study demonstrates that αBC-P can prevent cellular EMT, which is critical in PVR. However, a deeper understanding of the intricate details of metabolic reprogramming of TGFβ2-stimulated cells and the potential impact of αBC-P is needed to devise targeted interventions.

αB-crystallin plays an essential role in apoptosis inhibition, angiogenesis, and proteasome interactions, as well as being a molecular chaperone [8,19]. VEGF-A is one of the most potent angiogenesis stimulators and vascular permeability factors [56,57]. VEGF-A protein was increased in ocular tissues during angiogenesis and VEGF-A secretion into the blood was observed in CNV [19]. Between the two well-studied growth factors, VEGF-A and CTGF, our present study showed αBC-P suppressed CTGF expression but did not significantly alter VEGF-A expression. The reason for unchanged VEGF-A is unclear but may be linked to the difference in the experimental animal model and the time and frequency of αBC-ELP administration. The effect of dispase and treatment with αBC-ELP peptide on visual function showed improvement of c-wave by ERG with no appreciable changes in ‘a’ and ‘b’ waves. While this would suggest improvement of RPE function, further detailed work will be needed to explore the role of αBC-ELP in photoreceptor function. 

## 5. Conclusions

Our findings with TGFβ2-induced hRPE cells and dispase-induced PVR in mice show that αBC-P can block EMT from TGFβ2 treatment and that OXPHOS and glycolysis are involved in this process. Moreover, αBC-ELP was found to inhibit EMT from dispase treatment in vivo, and specific growth factors may be involved in this process. αBC peptide may have therapeutic potential in PVR by reversing the phenotype of EMT/MET and improving the mitochondrial function of RPE cells.

## Figures and Tables

**Table 1:** Primer Sequences Used for Real-Time RT-PCR.

Target Molecule	Accession Number	Forward Primer Sequence	Reverse Primer Sequence
αSMA	NM_001613	5′-TCTGTAAGGCCGGCTTTGC-3′	5′-TGTCCCATTCCCACCATCA-3′
Vimentin	NM_003380	5′-TGAGTACCGGAGACAGGTGCAG-3′	5′-TAGCAGCTTCAACGGCAAAGTTC-3′
E-cadherin	NM_004360	5′-ATTTTTCCCTCACACCCGAT-3′	5′-TCCCAGGCGTAGACCAAGA-3′
Sirt1	NM_012238	5′-TCCTGGACAATTCCAGCCATCTCT-3′	5′-TTCCAGCGTTATGTTCTGGGT-3′
GAPDH	NM_002046	5′-ACAGTCGCCGCATCTTCTT-3′	5′-CTTGATTTTGGAGGGATCTCGC-3′

αSMA; α-smooth muscle actin, Sirt 1; sirtuin 1 GAPDH; glyceraldehyde-3-phosphate dehydrogenase.

**Table 2:** List of Antibodies Used for Western Blot analysis.

Target Molecule	Antibody Type	Source	Dilution
αSMA (ab7817)	Mouse monoclonal	Abcam	1:1000
E-cadherin (24E10)	Rabbit monoclonal	Cell Signaling	1:1000
pSMAD2/3 ((S465/467)/(S423/425)	Rabbit monoclonal	Cell Signaling	1:1000
SMAD2/3 (D7G7)	Rabbit monoclonal	Cell Signaling	1:1000
Fibronectin (EP5)	Mouse monoclonal	Santa Cruz Biotechnology	1:1000
Vimentin (3CB2)	Mouse monoclonal	Santa Cruz Biotechnology	1:1000
SNAIL (C15D3)	Rabbit monoclonal	Cell Signaling	1:1000
SLUG (G-18)	Goat polyclonal	Santa Cruz Biotechnology	1:1000
CTGF (ab6992)	Rabbit monoclonal	Abcam	1:1000
GAPDH (MAB374)	Mouse monoclonal	EMD Millipore	1:1000

αSMA; α-smooth muscle actin, pSMAD2/3; phospho-smad2/smad3, SMAD2/3; smad2/smad3, CTGF; connective tissue growth factor, GAPDH; glyceraldehyde-3-phosphate dehydrogenase.

## Data Availability

All of the data is contained within the article and the Supplementary Materials.

## Supplementary Materials

The following supporting information can be downloaded at: https://www.mdpi.com/article/10.3390/antiox11102080/s1, Figure S1: Mitochondrial bioenergetics with TGFβ2 treatment of RPE cells.
