# Supplementary material for: Mechanisms of Epithelial-Mesenchymal Transition and Prevention of Dispase-Induced PVR by Delivery of an Antioxidant αB Crystallin Peptide
Source: Antioxidants (Basel). 2022 Oct 21;11(10):2080. doi: 10.3390/antiox11102080 (PMC9598590; doi:10.3390/antiox11102080)
Supplement: Supplementary file 1 [file antioxidants-11-02080-s001.zip › antioxidants-1877097-supplementary.pdf]

Figure. S1. Mitochondrial bioenergetics with TGFβ2 treatment of RPE cells

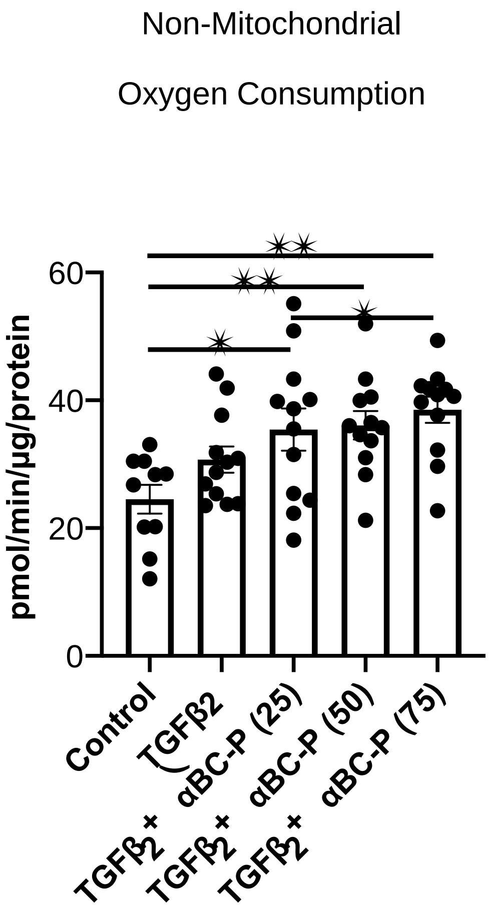

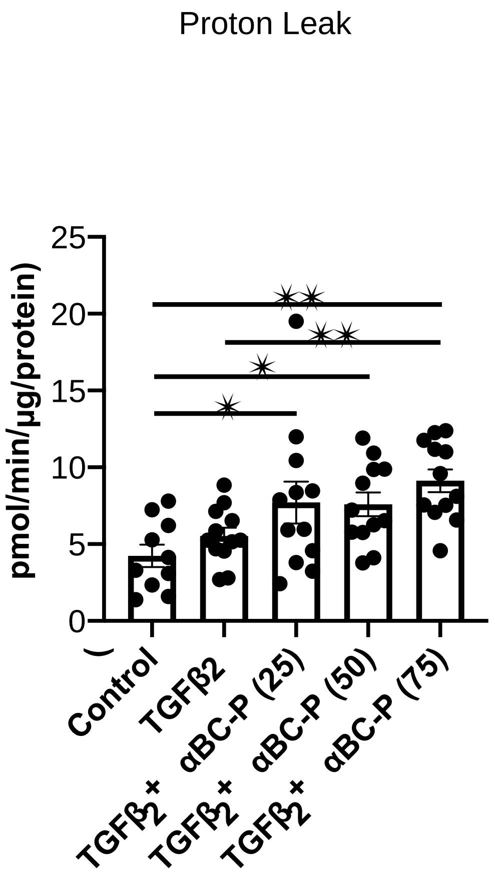

Mitochondrial bioenergetics were analyzed using Seahorse XFe96. Sub-confluent RPE cells were treated with TGFβ2 (10 ng/ml) alone or/with αBC-P (25, 50, and 75 μg/ml) in DMEM containing 3% FBS for 48 hours. TGFβ2-cotreated with αBC-P significantly increased non-mitochondrial oxygen consumption and proton leak, compared with the TGFβ2-treated group in a dose-dependent manner. αBC-P: αB crystallin peptide. Data normalized by μg/cellular protein. Values are means ± SEM. *$P < 0.05$, **$P < 0.01$. n=9-15.
